# Schiff Base‐UiO66 Composite‐based Dispersive Micro Solid‐Phase Extraction of Pesticides From Fruit Juices Combined With Dispersive Liquid‐Liquid Microextraction Prior to Gas Chromatography‐Flame Ionisation Detection

**DOI:** 10.1002/ansa.70102

**Published:** 2026-08-03

**Authors:** Behnam Hosseininezhad, Mahdi Bomorovat, Mahboob Nemati, Mir Ali Farajzadeh, Ramin Atazadeh, Mohammad Reza Afshar Mogaddam

**Affiliations:** ^1^ Food and Drug Safety Research Centre Pharmaceutical Sciences Institute Tabriz University of Medical Sciences Tabriz Iran; ^2^ Pharmaceutical Analysis Research Centre Pharmaceutical Sciences Institute Tabriz University of Medical Sciences Tabriz Iran; ^3^ Pharmaceutical and Food Control Department Faculty of Pharmacy Tabriz University of Medical Sciences Tabriz Iran; ^4^ Department of Analytical Chemistry Faculty of Chemistry University of Tabriz Tabriz Iran; ^5^ Engineering Faculty Near East University Mersin Turkey; ^6^ Department of Food Science and Technology Sou.C., Islamic Azad University Soufian Iran; ^7^ New Material and Green Chemistry Research Centre Khazar University Baku Azerbaijan

**Keywords:** dispersive liquid‐liquid microextraction, dispersive micro solid‐phase extraction, fruit juice, gas chromatography, metal‐organic framework, pesticide, Schiff base

## Abstract

A dispersive micro solid‐phase extraction method was introduced using a Schiff base‐UiO66 nanocomposite as the adsorbent in the extraction of multi‐class pesticide residues in pear, cherry, grape, and apricot juices. The extraction procedure was hyphenated with a liquid‐phase microextraction procedure, providing a high preconcentration factor and sensitive detection limit. During the extraction procedure, a small amount of the sorbent was added to the sample solution and dispersed via vortexing. Then, the sorbent particles were separated, and the adsorbed analytes were eluted by an appropriate water‐miscible organic solvent, which was subsequently used for the preconcentration of the analytes in the following microextraction step. Gas chromatography‐flame ionisation detector was utilised as a determination system. All effective factors were evaluated to obtain their optimum values, and then the method was validated. Low limits of detection (0.13–0.27 ng mL^−1^) and low limits of quantification (0.43–0.89 ng mL^−1^), acceptable extraction recoveries (59%–71%), high enrichment factors (295–355) and good relative standard deviations (≤10.1%) were the advantages of the method. Fortunately, application of the method on the studied samples showed that the fruit juices were free of the analytes.

## 1 Introduction

Pesticides are commonly used in modern agriculture to minimise the negative impacts of insects during cultivation and harvesting [1]. Pesticide usage in fruits is essential to combat pests that reduce farm production and also to enhance the quality of the fruits that reach consumers [2]. The most crucial concern related to pesticides is their residues, which are present in variable amounts in all types of agricultural products after harvest and cannot be controlled by consumers. Their toxic effects on human health are undeniable. Among the pesticides, several groups, including pesticides, fungicides, triazine herbicides and pyrethroids, have been identified in fruit juices [3]. Additionally, organophosphorus pesticides have been detected due to their high solubility in water, which is the main component of fruit juices [4]. Despite their positive effects, the use of pesticides should be done with caution [5]. European Union directives have regulated maximum residue limits for various pesticides in foods, water, etc. [6]. Chromatographic and separation approaches are recommended for accurate, precise and sensitive determination of pesticide residues in all kinds of foods. These methods are able to analyse different food toxins simultaneously [7]. Given that the sample preparation method significantly affects the results, various sample preparation techniques have been employed in the determination of toxins [8]. Various methods, including solid phase microextraction [9], dispersive micro solid‐phase extraction (DµSPE) [10], magnetic SPE [11] and dispersive liquid‐liquid microextraction (DLLME) [12, 13], have been utilised for the analysis of different pesticides from various matrices.

DµSPE is an adsorbent‐based extraction method widely used during the analysis of various compounds. This method offers significant advantages over the conventional SPE method, especially in terms of its simplicity and the use of various sorbents with tunable properties [14]. Various materials have been used as adsorbents in DSPE. The most effective of them is metal‐organic frameworks (MOFs) [15, 16]. Nanoparticles based on modified MOF have been widely utilised in DSPE methods. MOFs are compounds consisting of metal ions and organic ligands that have one‐, two‐, or three‐dimensional structures. These materials have high porosity, and because of their diverse pore structures, they can be used in extraction and material and energy storage processes [1]. In recent studies, the properties of MOFs have been improved by combining them with carbon‐based compounds to increase chemical stability, porosity, conductivity, and surface adsorption capacity [17]. Schiff base ligands are obtained from the condensation of primary amines with carbonyl compounds. Schiff base‐metal complexes have superior features such as ease of preparation, well‐defined solid‐state structures, chemical and thermal stability, and low cost. Also, they have been introduced as one of the main ligands in coordination chemistry [18, 19]. The major benefits of the DSPE method include low cost, high speed, simplicity, reproducibility, and wide applicability in analytical chemistry [20, 21]. However, DSPE has some drawbacks, too. One of the major problems is low enrichment factors (EFs) resulting from the use of large volumes of desorption solvent. Thus, a combination of DSPE with other methods like DLLME was examined to solve this problem [22, 23].

The main objective of this work was to develop a DSPE method using a Schiff base‐MOF nanocomposite to extract some pesticides from fruit juice samples and their analysis by gas chromatography‐flame ionisation detection (GC‐FID). To reach this goal, UiO‐66 modified by a Schiff base was used for the first time with the aim of extracting the analytes. The choice of UiO‐66 is justified by its properties such as high resistance to water and organic solvents and thermal stability up to 450°C in air [24, 25]. This work showed significant results by changing the MOF to UiO‐66‐NH_2_ to determine the concentration of pesticides in fruit juices.

## 2 Experimental

### 2.1 Chemical Compounds and Prepared Solutions

The pesticides studied included haloxyfop‐methyl ester, hexaconazole, diazinon, and diniconazole, all of high purity (>99%), and were bought from Dr Ehrenstorfer company (Augsburg, Germany). High‐performance liquid chromatography (HPLC)‐grade methanol and acetonitrile, as well as analytical‐reagent grade chemical compounds such as Zr(NO_3_)_3_, hydrochloric acid (37% w/w), acetone, sodium chloride, 2‐amino terephthalic acid, terephthaldehyde (TPA), *p*‐aminobenzoic acid (PABA), chloroform, sodium hydroxide, 1,1,2‐trichloro‐1,2,2‐trifluoroethane, carbon tetrachloride, dimethylformamide (DMF) and ethanol (99.8%) were all purchased from Merck (Darmstadt, Germany). Deionised water, provided by Ghazi Company (Tabriz, Iran), was utilised to prepare the working solutions for the optimisation and validation stages. A stock solution of the pesticides (50 mg L^−1^ of each) was prepared in methanol and stored at 4°C in a refrigerator. Working solutions were created by diluting the stock solution with deionised water.

### 2.2 Apparatus

To monitor and quantify the analytes under study, a GC‐FID system (Agilent 6890; Agilent Technologies, CA, USA) was employed. The injector had a sampling time of 60 s and a split ratio of 1:10, with temperatures set at 300°C for both the detector and injection port. Injections were manually performed using a 1‐µL microsyringe. The carrier gas (helium, purity ≥ 99.999%, Gulf Cryo, Dubai, UAE) was flowed at a rate of 1.0 mL min^−1^. To separate the analytes, an HP‐1 capillary column (95% dimethyl, 5% diphenyl poly‐siloxane with a film thickness of 0.25 µm) from Agilent, measuring 30 m × 0.25 mm i.d was utilised. The column oven was initially set to 80°C and, after running it was then ramped up to 300°C (at a rate of 8°C min^−1^ after 2 min. The column was kept at 300°C for 7 min. Additionally, for FID, hydrogen gas was generated by a hydrogen generator (GLAIND‐2200, Italy) at a flow rate of 30 mL min^−1^. The other systems used during characterisation and method optimisation are mentioned in Supporting Information.

### 2.3 Synthesis of Sorbent

First, UiO‐66‐NH_2_ MOF was prepared using a method reported in the literature [26]. To summarise, 0.182 g of Zr(NO_3_)_3_ and 0.134 g of 2‐amino terephthalic acid as a ligand were added to a mixture of 15 mL of DMF and 1 mL of concentrated hydrochloric acid. The solution was then transferred to an autoclave and placed in an oven at a temperature of 120°C for 24 h. Subsequently, centrifugation (6000 rpm for 6 min), washing (with 5 mL of DMF) and drying (at room temperature) steps were carried out on the crystals.

The Schiff base‐modified UiO‐66‐NH_2_ was prepared according to a method described in a previous study [19]. Specifically, 1.0 g of UiO‐66‐NH_2_ particles was dispersed in 100 mL of ethanol containing 0.5 g of TPA. Afterwards, refluxing was performed in an Erlenmeyer flask at 80°C for 6 h. It was then placed in a water bath thermostated at 80°C for an additional 6 h. The solid material was filtered, and to remove excess TPA, the solid was washed with ethanol. The obtained crystals were then dispersed into 100 mL of ethanol containing 0.5 g of PABA as a ligand and refluxed again for another 24 h. Finally, centrifugation, washing, and vacuum drying steps were performed, respectively.

### 2.4 DSPE‐DLLME Procedure

Five millilitres of deionised water containing the pesticides at a concentration of 50 ng mL^−1^ for each or diluted fruit juice (1:4 ratio with deionised water) was transferred into a 10‐mL glass test tube. Sodium chloride was dissolved in the solution at a concentration of 4% w/v NaCl. Then, the analytes were adsorbed by 15 mg of the sorbent with the aid of vortexing (for 4 min) to disperse the sorbent particles throughout the solution. After centrifugation, the sorbent settled at the bottom of the tube. The supernatant was then removed, and 1.25 mL of methanol was used to desorb the analytes with the aid of vortexing for 2 min. Following this step, the eluate was removed and mixed with 120 µL of chloroform. This mixture was rapidly dispersed into deionised water (5 mL) placed in a conical‐bottom glass test tube. Finally, the mixture was then centrifuged, and a portion of the collected phase (10 ± 0.5 µL) was injected into the GC‐FID system.

## 3 Results and Discussion

### 3.1 Characterisation of Sorbent

**FIGURE 1:**
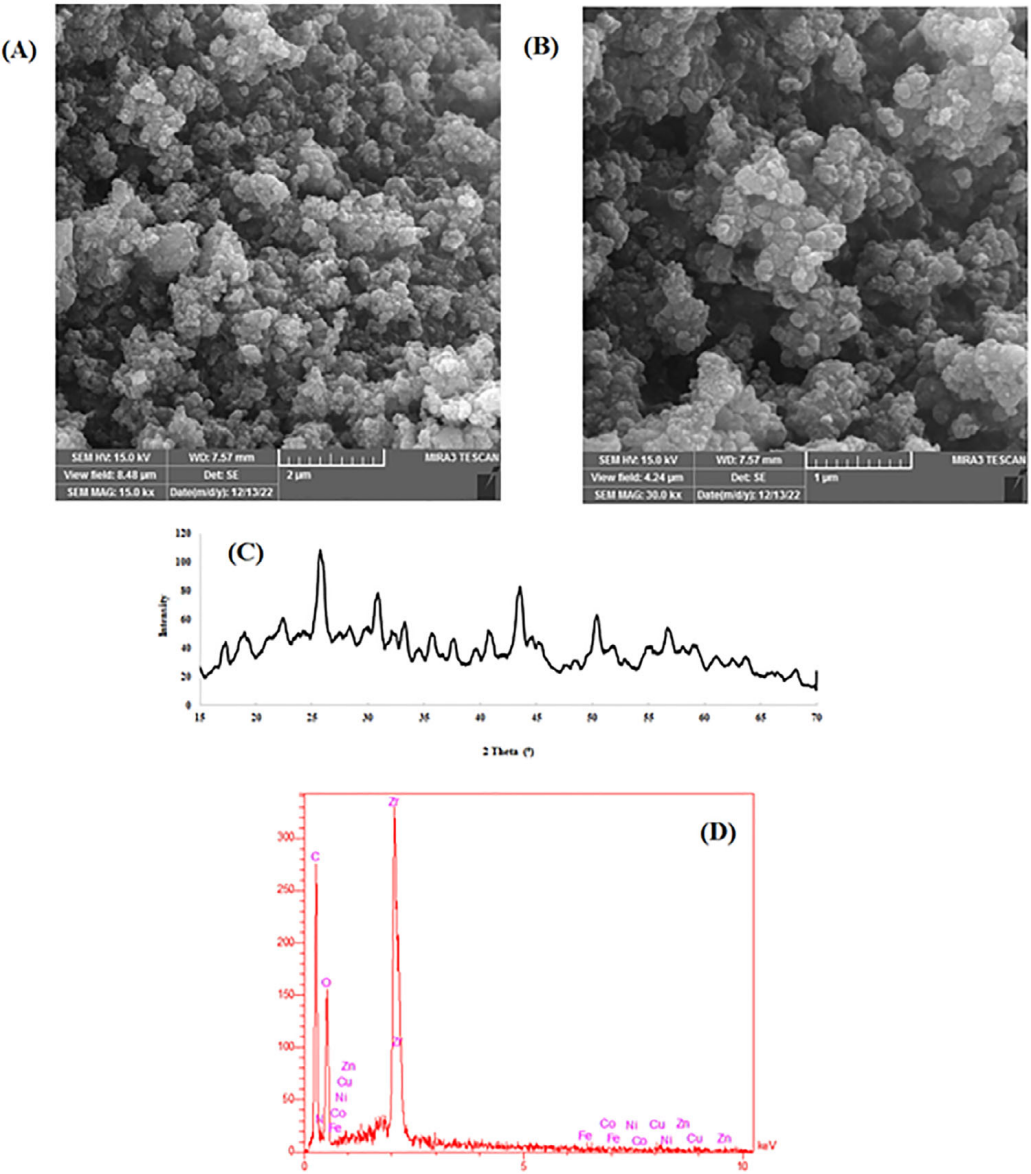
Scanning electron microscopy (SEM) images of metal‐organic framework (MOF) (A) before and (B) after modification, and X‐ray diffraction (XRD) patterns (C) and energy‐dispersive X‐ray spectroscopy (EDX) spectrum (D) of the sorbent.

The prepared Schiff base‐UiO66 composite was analysed using energy‐dispersive X‐ray spectroscopy (EDX), X‐ray diffraction (XRD) and scanning electron microscopy (SEM) techniques. Figure 1a,b shows the SEM images of the particles before and after modification, respectively. The spherical and nearly homogeneously dispersed particles are clearly visible in the images. EDX analysis was also performed on the sorbent in order to identify the elemental composition of MOF. According to Figure 1d, peaks associated with C, O, Zr, Fe, and N are observed with the weight percentages of 0.35%, 21.60%, 25.54% and 7.23%, respectively. XRD analysis was used to determine the crystal structure of the sorbent. The data indicate that the sorbent was successfully synthesised. The results shown in Figure 1c, the diffraction peaks at 25.7°, 30.8° and 43.5° are attributed to the crystal planes of the synthesised MOF.

### 3.2 Optimisation of Parameters Affecting Extraction Procedure

#### 3.2.1 Optimisation of Sorbent Amount

**FIGURE 2:**
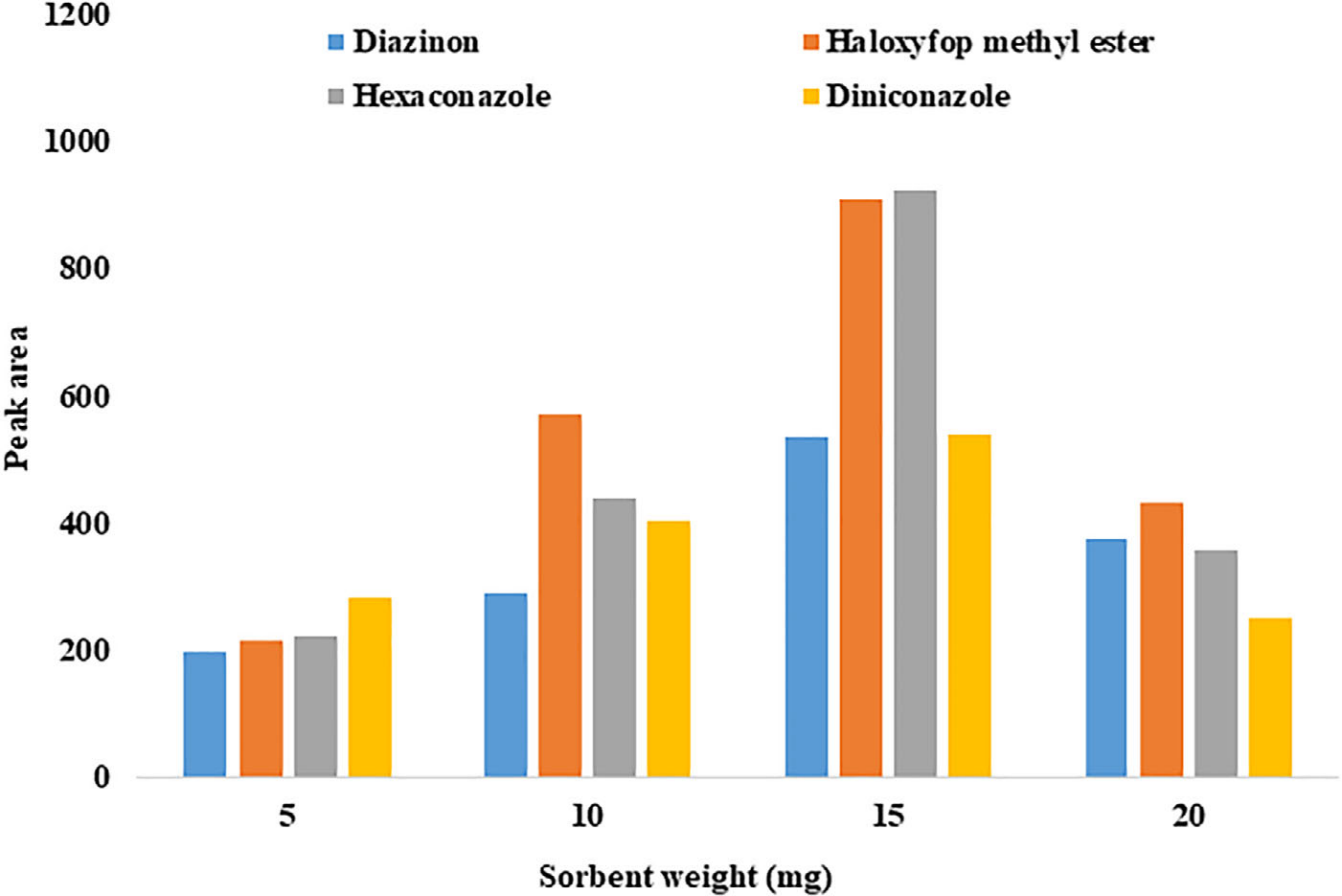
Effect of sorbent weight on the extraction efficiency of the method. Extraction conditions in dispersive solid‐phase extraction (DSPE) step, aqueous solution, 5 mL of deionized water containing 100 ng mL^−1^ of each analyte, adsorption time, 3 min; centrifugation time (speed), 5 min (6000 rpm), volume and type of elution solvent: 1.25 mL of methanol; and desorption time, 4 min; dispersive liquid‐liquid microextraction (DLLME) procedure, aqueous phase, 5 mL deionized water, volume and type of extraction solvent: 130 µL of chloroform, and centrifugation rate (time), 5000 rpm (5 min).

The amount of sorbent is a highly influential parameter in DSPE‐based methods, because it directly affects the adsorption of analytes. Therefore, the sorbent amount must be optimised. To check the effect of the amount of adsorbent, a series of tests was performed using 5, 10, 15 and 20 mg of the sorbent while keeping other parameters constant. The results, depicted in Figure 2, illustrate that the method's effectiveness increases up to 15 mg, but then decreases when the sorbent amount exceeds 15 mg. The results show that 15 mg of the sorbent is sufficient to adsorb the analyte. Hence, 15 mg of it was used in the next steps.

#### 3.2.2 Optimisation of Kind and Volume of Elution Solvent

**FIGURE 3:**
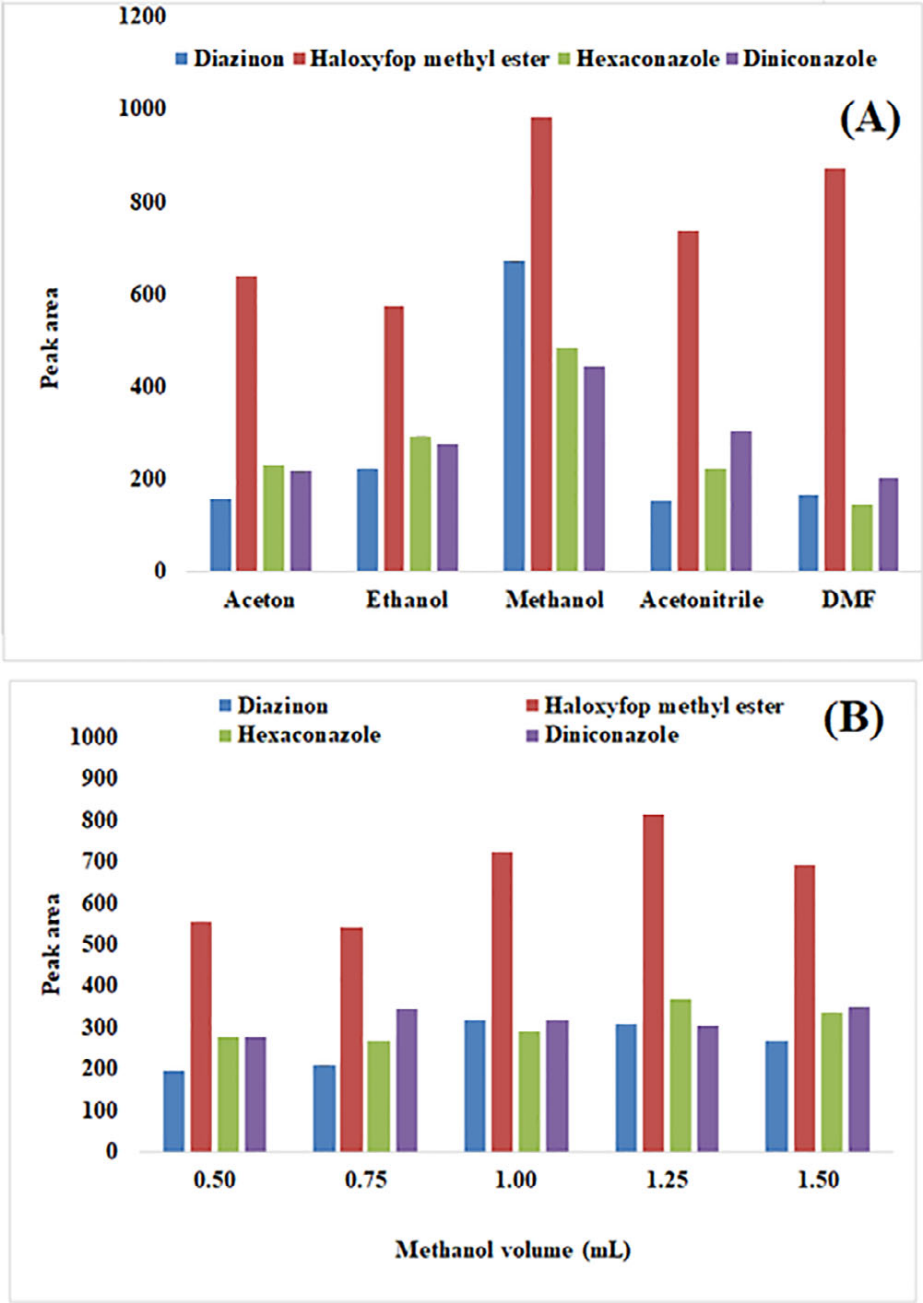
Choosing elution solvent type (A) and volume (B). Conditions: the same as those used in Figure 2, except for sorbent weight, which was 15 mg.

In this part, an elution solvent was utilised to desorb the analytes from the sorbent surface and also served as the dispersive solvent in DLLME. The efficiency of the method depends on the type of elution solvent, as different analytes have varying solubilities in various solvents. In this study, the selection of the elution solvent was investigated among acetone, methanol, ethanol, ACN and DMF due to their suitability for use in DLLME as a dispersive solvent. The data obtained in Figure 3A confirm the better efficiency of methanol among the solvents tested. Thus, methanol was opted for subsequent experiments.

To assess the effect of methanol volumes, volumes of 0.50, 0.75, 1.00, 1.25 and 1.50 mL were tested in the method. Different volumes of methanol can affect the desorption of the analytes from the sorbent surface and the dispersion of the extraction solvent used in DLLME into the aqueous phase. The data indicate that a volume of 1.25 mL of methanol provides the highest efficiency among the tested volumes (Figure 3b).

#### 3.2.3 Optimisation of Vortexing Time in Adsorption Step

**FIGURE 4:**
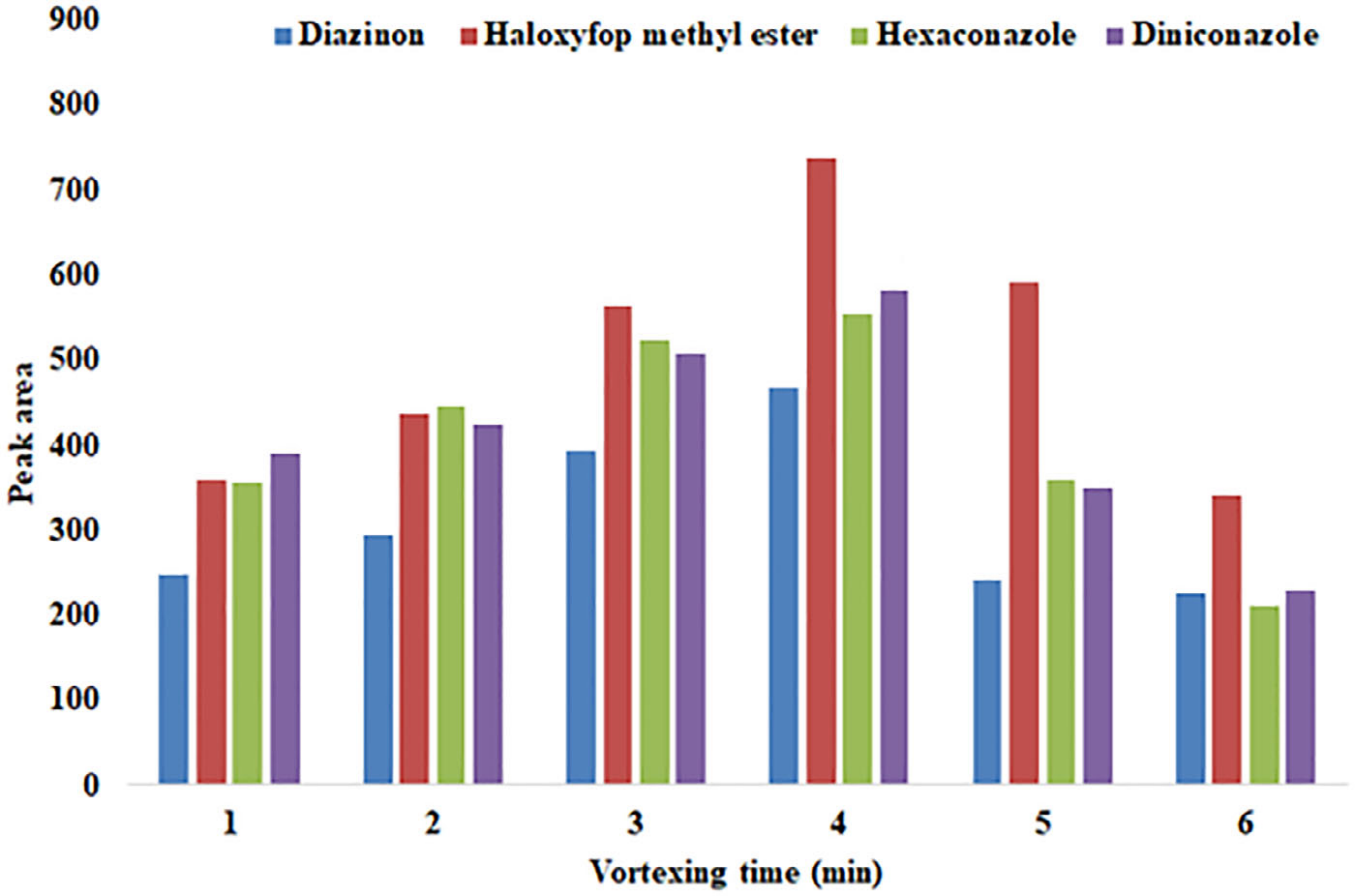
Optimising the effect of vortexing time on the efficiency of the method. Conditions: the same as those used in Figure 3, except 1.25 mL methanol was utilised.

Vortex agitation is a practical process used to increase the contact surface area between phases in various analytical methods. To evaluate the effect of this parameter on the method efficiency, different vortexing times (1–6 min) were tested. According to the obtained results (Figure 4), the peak areas of the analytes increase with vortexing time up to 4 min, after which they decrease. Therefore, a vortexing time of 4 min was selected.

#### 3.2.4 Study of Salt Addition Effect and Sample Solution pH

**FIGURE 5:**
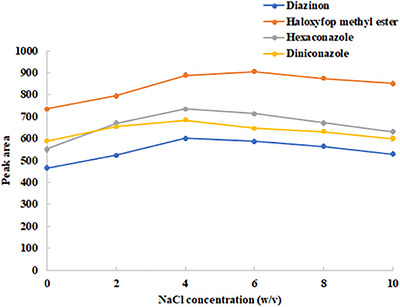
To evaluate the addition of salt on the efficiency of the method. Conditions were the same as those utilised in Figure 4, except a vortexing time of 4 was selected.

The effect of sodium chloride addition on the efficiency of the extraction method was investigated by dissolving different concentrations of sodium chloride in the range of 0%–10%, w/v. It is expected that salt addition decreases the solubility of analytes and enhances their tendency to adsorb onto the sorbent [27]. However, salt ions can occupy the pores and adsorption sites of the sorbent, which has a negative effect on the method efficiency. The experimental data (Figure 5) illustrate that salt addition has an increasing effect on the method efficiency up to 4% (w/v), followed by a decreasing effect. Thus, subsequent experiments were carried out in the presence of NaCl (4%, w/v).

Since the stability of the pesticides and the sorbent may vary at diverse pH values, the pH of the DSPE solution can be a significant parameter affecting the results of the method. In this study, to assess the effect of pH on the efficiency of extraction method, pH of the aqueous solution was adjusted at the pH of the aqueous solution was adjusted to pH values of 2, 4, 6, 8 and 10 using McIlvaine buffer (0.5 M). The obtained data showed that nearly constant analytical signals were obtained in the pH range of 4–8, and no pH adjustment was necessary because the pH of the samples fell within this range.

#### 3.2.5 Optimisation of Desorption Time

**FIGURE 6:**
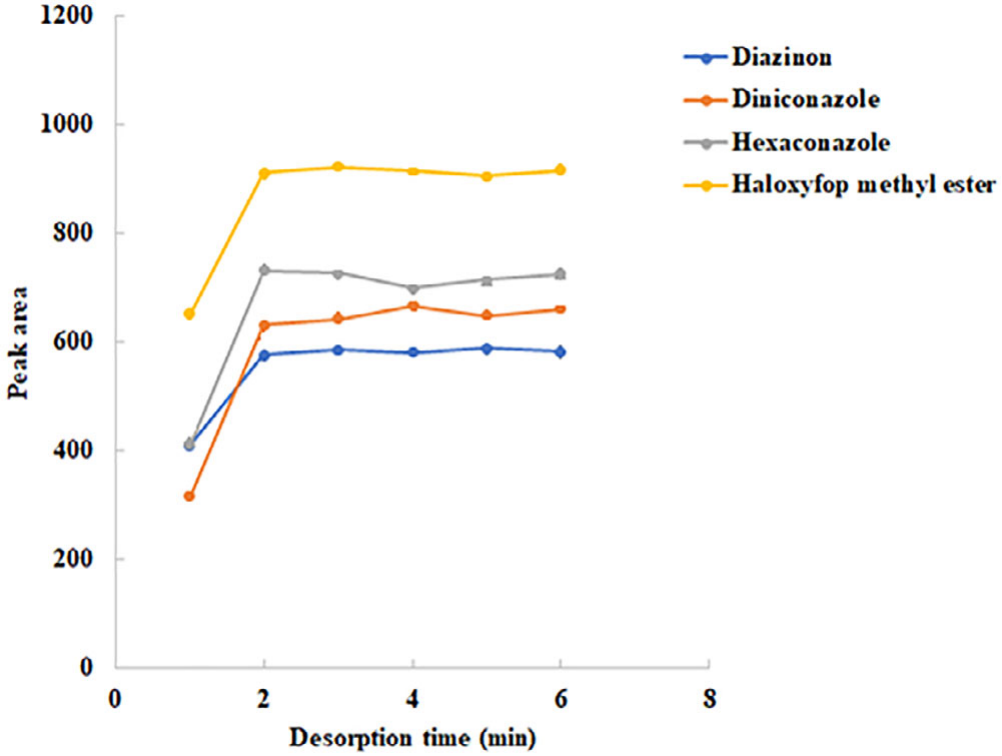
Optimising the effect of desorption time on the efficiency of the method. Conditions: the same as those used in Figure 5, except 200 mg of salt (4% w/v) was used.

Vortexing was also utilised to desorb the pesticides from the sorbent surface. In this study, the mixture of adsorbent and eluting solvent was vortexed for different durations (1‐6 min, in 1‐min intervals). The results (Figure 6) indicate that 4 min of vortexing is enough to elute pesticides from the sorbent surface.

#### 3.2.6 Selection of Extraction Solvent Type and Volume in DLLME

**FIGURE 7:**
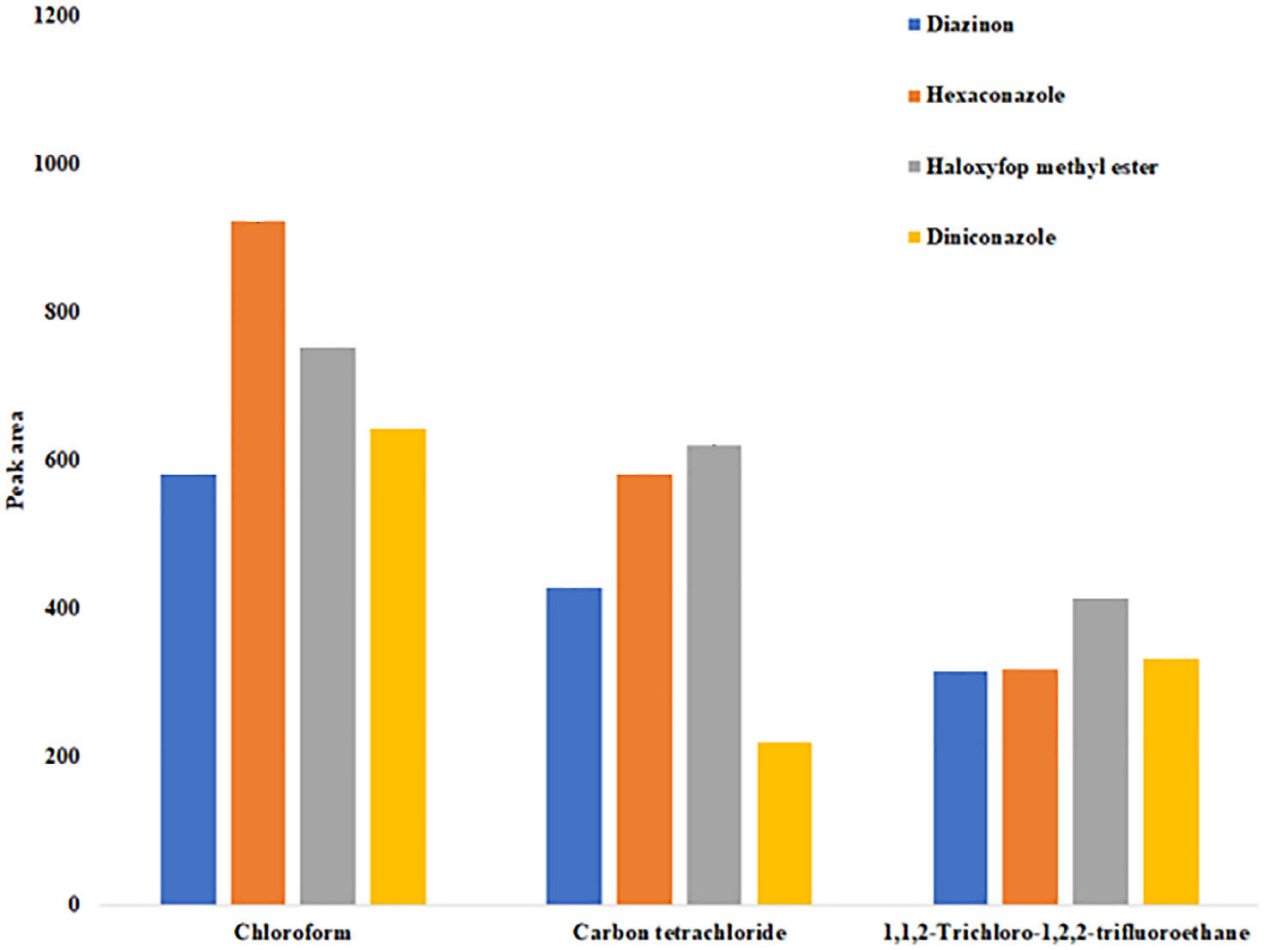
Selection of extraction solvent type in dispersive liquid‐liquid microextraction (DLLME). Conditions: The same as those used in Figure 6, except a desorption time of 4 min was chosen.

At this stage, a suitable solvent should be selected for the extraction, which has limited solubility in the aqueous phase. This is a fundamental step in the DLLME method. The chosen solvent also should possess low viscosity, good chromatographic behaviour, and a higher density relative to water. Accordingly, chloroform, carbon tetrachloride, and 1,1,2‐trichloro‐1,2,2‐trifluoroethane, which have the properties mentioned above, were evaluated. Based on the results in Figure 7, the highest analytical signals were obtained with chloroform; therefore, it was used in the next stage of optimisation. Then, optimisation of extraction solvent volume was performed, which directly affects the volume of the sedimented organic phase after centrifuging, limit of detection (LOD), EF, and extraction recovery (ER) of the analytes. The results obtained with different volumes (120, 130, 140 and 150 µL) showed that as the extraction solvent volume increased up to 130 µL, ERs of the analytes increased and then remained constant. Consequently, 130 µL of chloroform was used in the next steps.

#### 3.2.7 Salt Addition and pH Effect in DLLME

Another influential variable in the DLLME step is the pH of the aqueous phase, which has a significant effect on the extraction performance. The pH of the aqueous phase (deionised water) was adjusted to pH values of 2, 4, 6, 8 and 10 using 0.5 M NaOH or HCl solution. The results were high and nearly constant across the pH range of 4‐8. Additionally, the effect of NaCl concentration was evaluated by adding NaCl in the concentrations 0, 2, 4, 6%, 8%, and 10% (w/v); the obtained results were evaluated. The obtained results did not confirm any positive effect of increasing salt concentration in the aqueous solution. Therefore, neither salt addition nor pH adjustment was performed in subsequent experiments.

### 3.3 Adsorption and Desorption Mechanism

In this method, Schiff‐based UiO66 particles were used to adsorb the analytes from the sample solution. Multiple interactions can participate in the extraction step. The nitrogen and oxygen atoms of the sorbent can form hydrogen bonds with the electronegative atoms of the studied pesticides. Dipole–dipole and π–π stacking can occur by the conjugated system of the Schiff base and the aromatic linkers of the sorbent interacting with the aromatic and functional groups of the analytes. The Zr (IV) centres in UiO66 act as Lewis's acids, while the Schiff base nitrogen and oxygen atoms, as well as the electronegative sites on the analytes, serve as Lewis bases. In addition, hydrophobic interactions and occlusion may also contribute to the adsorption mechanism.

During the desorption procedure, the organic solvents used disrupt the interactions between the sorbent and analytes by solvating the analytes more strongly than surface adsorption interactions. Also, the organic solvents reduce the electrostatic and Lewis's acid–base interactions among the pesticides and the sorbent sites, eluting the analytes.

### 3.4 Analytical Parameters

At this stage, following US Food and Drug Administration protocols, the quantitative performance of the method was evaluated under optimal conditions. The evaluated parameters included LOD (signal (S)/noise (N) = 3), limit of quantification (LOQ, S/N = 10), ER, linear range (LR) of the calibration curve, EF, coefficient of determination (*r* ^2^), and relative standard deviation (RSD) were evaluated in the optimal conditions. The results are summarised in Table 1. The data show that the LODs and LOQs are in the ranges of 0.13–0.27 and 0.43–0.89 ng mL^−1^, respectively. The RSDs for intra‐ and inter‐day precisions were in the ranges of 5.1%–9.0% and 5.4%–10%, respectively. ERs (ER% = $\frac{n_{sed}}{n_{0}}$ × 100, n_0_: total amount of analyte and n_sed_: the amount of analyte migrated into the sedimented phase) were between 59% and 71%. The EF values ranged from 295 to 355. These data indicate that this method is effective for the analysis of the studied pesticides at low concentrations with high efficiency.

**TABLE 1:** Quantitative features of the developed method.

					RSD %^e^							
					Intra‐day precision at the concentrations of			Inter‐day precision at the concentrations of				
Analytes	LOD^a^ (ng mL^−1^)	LOQ^b^ (ng mL^−1^)	LR^c^ (ng mL^−1^)	*r* ^2^ ^d^	1 (ng mL^−1^)	10 (ng mL^−1^)	100 (ng mL^−1^)	1 (ng mL^−1^)	10 (ng mL^−1^)	100 (ng mL^−1^)	ER ± SD^f^ (%)	EF ± SD^g^
Diazinon	0.21	0.69	0.69–250	0.998	7.8	6.8	6.2	8.2	7.2	6.5	68 ± 4	340 ± 20
Hexaconazole	0.27	0.89	0.89–250	0.997	8.9	7.2	5.5	9.0	7.5	6.2	59 ± 3	295 ± 15
Haloxyfop methyl ester	0.17	0.56	0.56–250	0.999	9.0	5.3	5.1	10.1	6.8	5.4	71 ± 4	355 ± 20
Diniconazole	0.13	0.43	0.43–250	0.999	8.9	6.4	5.2	9.5	6.8	6.5	70 ± 3	350 ± 15

^a^ Limit of detection (S/N = 3).

^b^ Limit of quantification (S/N = 10).

^c^ Linear range.

^d^ Coefficient of determination.

^e^ Relative standard deviation for intra– (*n* = 6) and inter–day (*n* = 4) precisions.

^f^ Extraction recovery ± standard deviation (*n* = 3).

^g^ Enrichment factor ± standard deviation (*n* = 3).

### 3.5 Real Samples Analysis

Following the optimisation and validation stages, the applicability of the prepared sorbent and the proposed approach for the analysis of the studied pesticides were investigated through the analysis of different fruit juice samples including pear, grape, cherry and apricot. Fruit juices were purchased from a local supermarket in Tabriz (East Azerbaijan, Iran) and then they were diluted with deionised water at a ratio of 1:4. The obtained chromatograms showed no peaks eluting at the retention times of the analytes. To evaluate the matrix effect of the samples on the efficiency of the method, all samples were spiked at two concentrations (5 and 50 ng mL^−1^, each analyte) and the method was applied after dilution with deionised water (1:4 ratio). As presented in Table 2, the obtained mean relative recoveries were in the range of 91%–101%. These results indicate that there is no significant matrix effect in the fruit juices after dilution. Therefore, the synthesised sorbent can be considered a suitable and efficient sorbent for the extraction of the target analytes from real samples such as fruit juices.

**TABLE 2:** Results of the relative recoveries to check matrix effect of the samples in determination of the studied analytes.

Analyte	Mean relative recovery ± standard deviation (*n* = 3)			
	Pear	Cherry	Grape	Apricot
All samples were spiked with each analyte at a concentration of 5 ng mL^−1^				
Diazinon	95 ± 3.2	92 ± 4.0	96 ± 5.0	9 ± 4.5
Hexaconazole	95 ± 2.1	95 ± 5.4	95 ± 5.1	94 ± 3.2
Haloxyfop methyl ester	93 ± 4.2	94 ± 6.5	95 ± 2.4	95 ± 2.2
Diniconazole	92 ± 3.3	96 ± 2.1	94 ± 1.6	92 ± 4.3
All samples were spiked with each analyte at a concentration of 50 ng mL^−1^				
Diazinon	94 ± 1.9	98 ± 4.0	95 ± 3.2	96 ± 3.4
Hexaconazole	95 ± 3.0	95 ± 3.0	91 ± 4.1	95 ± 2.1
Haloxyfop methyl ester	91 ± 3.8	99 ± 6.9	92 ± 2.0	92 ± 1.6
Diniconazole	93 ± 3.5	101 ± 6.2	98 ± 2.4	97 ± 4.2

### 3.6 Comparison of the Method With Other Approaches

All quantitative characteristics of the method (RSD, LR, LOD, LOQ, EF and ER) were compared with those of other methods published in previous studies for the analysis of the selected analytes (Table 3). The RSD results were reported as comparable to or better than those of other studies. The LODs achieved in the present study are better than the other studies, except when LC‐tandem mass spectrometry was used as the detection system, which is inherently more sensitive than GC‐FID. Similarly, the LOQs are lower than those reported in other studies. As is evident from the results, the EFs of this method are higher than or comparable to those of the other methods. Also, this method has comparable LRs relative to the other mentioned methods. Overall, these results demonstrate that the present method is a fast, easy, sensitive, and reliable analytical method and can be effectively used to measure pesticide residues in fruit juices.

**TABLE 3:** Comparison of the developed method with other methods.

Method	Sorbent type (amount)	Sample	LOD^a^ (ng mL^−1^)	LOQ^b^ (ng mL^−1^)	LR^c^ (ng mL^−1^)	RSD^d^ (%)	EF^e^	ER^f^ (%)	Refs.
DSPE‐ LC‐MS/MS^g^	NH_2_‐UiO‐66(Zr) MOF (7 mg)	Fruit juices	0.02–0.10	0.3–1.4	0.29–500	≤ 8.8	37–45	74–89	[28]
DSPE‐ GC‐MS^h^	UiO‐66 (50 mg)	Organic and conventional vegetables	0.4–2.0	0.49–1.2	10–500	≤14.6	—	—	[29]
DSPE‐DLLME–GC–FID^i^	ZIF67‐CDs (40 mg)	Fruit juices	0.22–0.72	0.73–2.40	2.40–1000	≤ 5.8	375–445	75–89	[30]
DSPE‐DLLME‐GC‐FID^i^	NH_2_‐Uio‐66(Zr) (15 mg)	Fruit juices	0.13–0.27	0.43–0.89	0.89–250	≤ 9.0	295–355	59–71	This work

^a^ Limit of detection (ng mL^−1^).

^b^ Limit of quantification (ng mL^−1^).

^c^ Linear range (ng mL^−1^).

^d^ Relative standard deviation.

^e^ Enrichment factor.

^f^ Extraction recovery.

^g^ Dispersive solid‐phase extraction–liquid chromatography‐tandem mass spectrometry.

^h^ Dispersive solid‐phase extraction–gas chromatography‐mass spectrometry.

^i^ Dispersive solid‐phase extraction—dispersive liquid–liquid microextraction ‐gas chromatography‐flame ionisation detection.

## 4 Conclusions

One of the most important safety indicators for fruits and vegetables is the control of pesticide residues. Consequently, the development of a sensitive and efficient extraction approach has received considerable attention. In this study, a DµSPE‐DLLME method was introduced to preconcentrate and extract various pesticides such as hexaconazole, diazinon, diniconazole, haloxyfop methyl ester and hexaconazole from fruit juice samples before their specification by GC‐FID. Low amounts of the sorbent can extract the analytes quantitatively. The acceptable extraction efficiency of the method can be related to the high porosity of the sorbent and the good ability of the DLLME in the enrichment of the analytes. The developed method offers several advantages, including extensive linearity, good reproducibility, low RSDs, high EFs, ease of operation and negligible matrix effect, making it suitable for the determination of trace levels of pesticides at ng mL^−1^ in fruit juices. In addition to the analytical capability of this method, the developed method has wider implications for food safety monitoring and public health protection. The method can support regulatory compliance and routine quality control in food industries. Also, the low consumption of organic solvents and sorbent aligns with green chemistry principles, reducing the environmental footprint of analytical laboratories. Future work could focus on establishing the range of analytes, automating the extraction procedure, and validating the method for routine use in official food control laboratories.

## Author Contributions

**Behnam Hosseininezhad**: conceptualisation, investigation, writing – original draft, validation, methodology and formal analysis. **Mahdi Bomorovat**: conceptualisation, investigation, writing – original draft and resources. **Mahboob Nemati**: conceptualisation, investigation, writing – original draft, methodology and resources. **Mohammad Reza Afshar Mogaddam**: conceptualisation, investigation, funding acquisition, methodology, validation, formal analysis, resources and supervision. **Mir Ali Farajzadeh**: writing – original draft, methodology, validation, formal analysis and resources. **Ramin Atazadeh**: conceptualisation, writing – original draft, methodology, validation and resources.

## Funding

This study was supported by grants from Tabriz University of Medical Sciences under grant number 70454 and ethical code IR.TBZMED.VCR.REC.1401.316.

## Conflicts of Interest

The authors declare no conflicts of interest.

## Use of Generative AI and AI‐Assisted Technologies in the Writing Process

AI‐assisted technologies declaration During the preparation of this manuscript the authors used Quillbot version 4.49.0 and deepseek to improve the language level and paraphrase of the text. After using this service, the authors reviewed and edited the content as needed and take full responsibility for the content of the published article.

## Supporting information




**Supporting File**: ansa70102‐sup‐0001‐SuppMat.docx.

## Data Availability

The data that support the findings of this study are available from the corresponding author upon reasonable request.
