# Supplementary material for: Schiff Base‐UiO66 Composite‐based Dispersive Micro Solid‐Phase Extraction of Pesticides From Fruit Juices Combined With Dispersive Liquid‐Liquid Microextraction Prior to Gas Chromatography‐Flame Ionisation Detection
Source: Anal Sci Adv. 2026 Aug 3;7(2):e70102. doi: 10.1002/ansa.70102 (PMC13432960; doi:10.1002/ansa.70102)
Supplement: Supplementary file 1 — Supporting File: ansa70102‐sup‐0001‐SuppMat.docx. [file ANSA-7-e70102-s001.docx]

**Supplementary data**

**Schiff base-UiO66 composite based dispersive micro solid phase extraction of pesticides from fruit juices combined with DLLME prior to GC-FID**

Behnam Hosseininezhad ^1^, Mahdi Bomorovat^2^, Mahboob Nemati ^1,3^, Mir Ali Farajzadeh ^4,5^, Ramin Atazadeh^6^, Mohammad Reza Afshar Mogaddam ^1, 7*^

*^1.^ Food and Drug Safety Research Center, Pharmaceutical Sciences Institute, Tabriz University of Medical Sciences, Tabriz, Iran*

*^2^ Pharmaceutical Analysis Research Center, Pharmaceutical Sciences Institute, Tabriz University of Medical Sciences, Tabriz, Iran*

*^3.^ Pharmaceutical and Food Control Department, Faculty of Pharmacy, Tabriz University of Medical*

*Sciences, Tabriz, Iran*

*^4.^ Department of Analytical Chemistry, Faculty of Chemistry, University of Tabriz, Tabriz, Iran*

*^5.^ Engineering Faculty, Near East University, 99138 Nicosia, North Cyprus, Mersin 10, Turkey*

*6 Department of Food Science and Technology, Sou.C., Islamic Azad University, Soufian, Iran*

*^7^ New Material and Green Chemistry Research Center, Khazar University, 41 Mehseti Street, Baku AZ1096, Azerbaijan*

*Corresponding authors:

Dr. M.R. Afshar Mogaddam

E–mail addresses: [mr.afsharmogaddam@yahoo.com](mailto:mr.afsharmogaddam@yahoo.com)

Tel.: +98 4131772354

*Instrumentation*

pH adjustments were made using a Metrohm pH meter model 654 (Herisau, Switzerland). An L46 vortex (Labinco, Breda, the Netherlands) was utilized for mixing. The centrifuge used for phase separation during extraction was Hettich, model D-7200 (Kirchlengern, Germany). A magnetic heater stirrer, model MR 3001K (Heidolph, Germany) was also utilized in the preparation of MOF. A Sartorius analytical balance (±0.0001 g) (Gottingen, Germany), a TV3U oven Shahla Co. (Tehran, Iran), and an ultrasonic bath (Backer, Vcleaner, Iran). The X-ray diffraction (XRD) pattern of the synthesized sorbent was gained using a Siemens D500 diffractometer (Siemens AG, Karlsruhe, Germany) at a voltage of 35 kV with a scanning speed and range of 1°min^-1^ and 4-73, respectively. The surface features of the sorbent were studied by scanning electron microscopy (SEM) (Tescan, Mira3).
